# Paying clinicians to join clinical trials: a review of guidelines and interview study of trialists

**DOI:** 10.1186/1745-6215-10-15

**Published:** 2009-03-10

**Authors:** James Raftery, Christine Kerr, Sheila Hawker, John Powell

**Affiliations:** 1 Wessex Institute for Health R&D, School of Medicine, University of Southampton, Southampton, UK; 2 Department of Epidemiology and Public Health, Medical School, University of Warwick, Coventry, UK

## Abstract

**Background:**

The motivations of clinicians to participate in clinical trials have been little studied. This project explored the potential role of payment for participation in publicly funded clinical trials in the UK. The aims were to review relevant guidelines and to collate and analyse views of clinical trialists on the role of payments and other factors that motivated clinicians to join clinical trials.

**Methods:**

Review of guidelines governing payments to clinicians for recruitment to trials. Semi-structured interviews with a range of NHS clinical trial leaders, analysed using qualititative methods.

**Results:**

While UK guidelines had little to say specifically on payments linked to recruitment, all payments have become highly regulated and increasingly transparent. Interview participants believed that expenses arising from research should be covered. Payments in excess of expenses were seen as likely to increase participation but with the risk of reducing quality. Motivations such as interest in the topic, the scope for patients to benefit and intellectual curiosity were considered more important. Barriers to involvement included bureaucracy and lack of time.

**Discussion:**

Limited scope exists for paying clinicians over-and-above the cost of their time to be involved in research. Most trialists favour full payment of all expenses related to research.

**Conclusion:**

Payment of clinicians beyond expenses is perceived to be a less important motivating factor than researching important, salient questions, and facilitating research by reducing bureaucracy and delay.

## Background

Most clinical trials in the UK fail to meet their recruitment targets [1]. An analysis of multicentre trials funded by HTA and MRC showed that 45% failed to reach 80% of target.[2] Only 10% of eligible patients were recruited in a review of one trial [3]. Less than half of participating clinicians succeeded in recruiting any patients in two other trials[4,5]. Commercial trials report similar problems with 30% of sites failing to recruit a single patients and 70% failing to meet agreed recruitment targets [6]. Studies that recruit too few patients might not only miss clinically important effects, but raise ethical questions about exposing volunteers to new treatments in inconclusive research. Slow recruitment can increase the cost of trials and delay the production of important evidence. Identified barriers included: time constraints, lack of staff and training, worries about the doctor-patient relationship, concern for patients, loss of professional autonomy, consent procedures and the lack of reward and recognition [1,7]. A systematic review [8] of incentives for participation indicated the importance of personal links between lead researcher and collaborating clinicians. Another systematic review [9] of the role of payments for participation located only three studies, none from the UK, all indicating that payment was of minor importance. All of these reviews recommended further research.

This study had three objectives:

• To outline current UK practice regarding the payment of financial incentives to healthcare professionals for recruitment of patients to trials.

• To explore the attitudes, beliefs and behaviour of healthcare researchers in relation to financial incentives for recruitment to trials.

• To indicate how financial incentives are viewed in relation to other barriers and facilitators to healthcare professionals recruiting patients to clinical trials.

Financial incentives include paying individual recruiters in cash, such as per recruit enrolled, or reimbursing to the practice or trust to cover additional costs associated with participation in a trial [10]. Gifts can be seen as equivalent to cash payments.

A fuller account of this research, funded by the NHS HTA Programme has been published [11] and is available online.

## Methods

To meet the first objective searches of bibliographic databases, and direct inquiries to key UK agencies and searches of their websites were undertaken in June 2006 to identify UK guidelines related to payment of clinicians in research. We also undertook web searching using Google. The database search was carried out in parallel with a literature review of studies examining the effectiveness of incentives and we looked for guidelines during the inclusion/exclusion process of the effectiveness review [11]. The UK agencies included the major research funders (such as the Medical Research Council and Wellcome Trust), professional organisations (such as the Association of the British Pharmaceutical Industry and the British Medical Association), and other research stakeholders such as the Central Office for Research Ethics Committees.

To meet the other objectives, semi-structured interviews were undertaken with researchers who had led clinical trials. A draft interview schedule was refined after eight pilot interviews [11]. In the schedule questions were structured around two main themes: the motivations for taking part in clinical trials (their personal motivations and their views of other's motivations); and their experience of and attitudes towards the use of incentives for recruitment. Approval for the study was granted by the Southampton and South West Hampshire Research Ethics Committee.

Researchers were selected to reflect diversity in five dimensions:

• Geographical location

• Primary and secondary care settings

• Clinicians and non-clinicians

• A range of specialties

• Currently active and inactive researchers

Lead investigators in ongoing trials in April 2006 funded by the NHS HTA Programme were identified from the public database Current Clinical Trials [11]. Of these 38 (27 doctors, 2 nurses and 9 non-clinical) were invited to participate. Six other clinicians with research experience who were no longer active in research were interviewed, having been identified by snowball sampling. All the active researchers approached agreed to be interviewed. Two thirds of inactive researchers identified through snowball sampling refused (six agreed, 12 refused).

All 44 interviews took place at the respondents' place of work between September 2005 and April 2006. All were audio- recorded and fieldnotes were made. Two investigators (CK and SH) individually undertook line-by-line open coding of all transcripts to identify empirically grounded [13]descriptive labels for phenomena related to the research aims. After comparison and discussion an agreed system of coding was devised and applied, approved by the wider research team who each read a sample of transcripts. NVIVO was used to assist data management and analysis [14].

## Results

### Review of guidelines

The review of guidelines on payment for involvement in clinical trials indicated that NHS organisations must charge the full cost for commercial trials [15], using the model Clinical Trial Agreement [16] unless good reasons exist to the contrary. Payment should be to the relevant NHS trust rather than to individual clinicians [14] and could be in the form of an amount per patient recruited [16]. Ethics committee approval, which is mandatory [17], requires submission of full information on all payments to researchers and patients. General Medical Council guidelines [18] require doctors engaged in research to disclose to patients entering trials how the research is funded and of any benefits to them or their departments.

Two points were striking: first that the guidelines said little about payments to individuals and secondly, what was said tended to focus on commercially funded trials. The Association of British Pharmaceutical Industries [19], Medical Research Council [20], British Medical Association [21] and Department of Health guidelines [22,23] did not mention payments to individual clinicians. The General Medical Council guidelines [18] were the most emphatic in requiring transparency on all payments but provided no indication of what levels of payments might be appropriate.

### Interview study

The semi-structured interviews explored the issue of payment for participation in trials and the relative importance of other motivating factors. The participants included thirteen primary care clinicians, fourteen secondary care clinicians, two other healthcare professionals, nine non-clinical researchers, and six non-research-active clinicians. The following themes were identified:

### Expenses versus incentives

Regarding payments, the interviews found that participants drew a strong distinction between expenses and payment over-and-beyond expenses. The former were strongly supported while the latter were not. This reimbursement was felt to be particularly important in undertaking research in primary care. As this respondent noted, *"I think the key thing if you're doing a trial in primary care now, if you don't offer any money then it's not going to happen" *(HR6).

### Positive effects of incentives

Payments were seen as both improving recruitment and a way of acknowledging the work of others, valuing their time and showing respect. *"If people feel respected and their time valued, they will do a lot more for it" *(GP3). Some lead researchers were already paying collaborators an amount per patient recruited, based on the estimated cost. This was seen as proportional, rewarding effort and enabling quality control. A related topic was the idea of a market economy in research participant recruitment and the need (particularly in primary care) to compete with pharmaceutical industry funded trials. *"The money falling where the patient is is important to ensure recruitment. There's no doubt if we had, for example, offered an incentive of a certain number of pounds to a service we're delivering x number of patients that would have improved recruitment without a doubt. With the ability to penalise the people if they don't" *(HD2).

### Negative effects of incentives

Many thought that while paying clinicians improved recruitment it could reduce quality. *"I mean indeed the more you get paid the more you're going to... the more the impetus is to act unethically. You know, theoretically if I'm getting 10 grand per patient as opposed to £10, I want to give them the bloody disease, you know" *(HD11). Concerns were raised about payments creating conflicts of interest. *"I think there is a bit of a conflict of interest over what's going to be best for the patient what's going to be best for my bank balance" *(GP13). Interviews also revealed worries about possible reductions in the rigour of research, with the possible threats of ineligible or coerced participants being recruited, or even fraud in the form of imaginary patients. *"I think the problems clearly are fraud, of misuse of the system as there are with any form of benefit and I think fraud is the main one, either if patients are getting money, fraud on their part, or fraud from doctors, nurses or abuse of the system" *(GP8). Some interviewees feared that financial payments in excess of costs could erode altruism and encourage 'unsuitable' researchers who would provide lower quality research.

### Principles for payments

Interviewees suggested principles for payments: good study design, payments to organisations rather than individuals, and improved transparency and disclosure. *"There are a number of elements for good practice in that, one is not over-recruiting from any one centre, another is making sure that you assess centres properly and making sure that the people there are trained properly and all those things reduce the potential for bad practice. But you have a place that's well organised and you are sort of giving them money that recompenses their, that covers their expenses, I think it is unlikely to be a place that is going to run into ethical issues" *(HR5).

*"There needs to be a clear process, explicit process as to why it's happening, where the benefit is being seen. I think there is a significant danger in an individual receiving money... And that's the major one I think" *(HD2).

The following probity tests were suggested:

*"If you are embarrassed about the amount of money you are being paid for the job you are doing, then you are being paid too much. You know, if you are being paid £500 for recruiting this person into this study, and you're going to be embarrassed about that – well you are asking too, you shouldn't be doing it" *(GP10).

*"I think the ultimate test I've always applied is "(Are you), would you be comfortable discussing [payments] with a patient?"*(HD8).

### Motivating factors for research participation

The most prominent factors that encouraged research participation, were: interest in the research question, intellectual curiosity and potential benefit to patients (including access to treatments or drugs, and closer monitoring). For example, *"I think it's important ... It needs to be quite concise and it will ring people's bell. Some topics are interesting to certain people and they are more likely to recruit to those" *(HD7).

*"Well certainly the evidence suggests that patients who go into trials get better health care. That's certainly, well maybe not evidence, but it's certainly the anecdote, I believe there probably is some evidence there, but I haven't looked at it ...You get, well that's something, you get more attention. And therefore if you get more attention one assumes you get better health care overall" *(HR5). Against this they noted the risk that patients could be randomised to a non-treatment group.

Less prominent motivating factors included altruism and career progression. Most believed altruism was a diminishing factor because of increased reliance on money and increased regulation of trials. Many thought all clinicians should be required to take part in research. Recognition and enhancement of reputation were a further minor theme but again were not seen as primary motivating factors. Likewise publication authorship was not seen as a prominent motivation. Publicly-funded trials were thought preferable to those funded by the pharmaceutical industry, with many spontaneous criticisms of the latter.

### Barriers to taking part in clinical trials

Lack of time was the main reason given for not taking part in trials. *"...but what happens is you kind of join up and don't foresee just quite how much time commitment it is and then you're in, even with the best will in the world, in your surgery and unless I've got spaces I just don't have the time to do it or else I will put all my patients back half an hour and you don't want to do that ... So I didn't recruit any patients and it was a time issue" *(GP13). Trials, many suggested, should be designed to cause the minimum disruption to clinical practice. Research time had to compete with targets in the new consultants' and GP contracts. Bureaucracy was seen as a major barrier to research involvement. The EU Directive on Good Clinical Practice, the Data Protection Act, and NHS research governance were all criticised as unduly restrictive. *"I think it's [research] become much, much more difficult to do as such because of research governance. I think that a lot of people in the NHS are completely baffled by research governance and they're terrified of doing the wrong thing" *(HR1).

### Contextual factors

The culture of the NHS was seen as prioritising clinical work over research but for some clinicians research provided a welcome alternative. The need for a culture that valued research within the NHS was widely stated. *"So yes we need an NHS that is completely research conscious, wanting research to happen but that doesn't mean that everybody needs to be a researcher" *(HR2). Training in research, it was suggested, should start before clinicians got 'bogged down' by clinical work and private practice. Good communication within research projects was seen as essential: this meant keeping collaborators informed, building rapport and creating good working relationships. "*(We've) always had very charismatic, well organised people doing the day-to-day trial management, and I think, you know, if you've got somebody who can go in and talk to the practices, be receptive to their problems, problem solve, then that seems to work" (GP7)*.

Clinical specialties were seen as differing in their research-friendliness, with surgery posing particular difficulties. *"Surgery's quite an interesting area to do trials because it's very difficult to persuade surgeons about uncertainty and exploring uncertainty, so in the past we've tended to do cohort studies" *(HD4). General practice differed in continuing to offer scope for payments to individual clinicians rather than to employing trusts. Few differences were found by geographical location, between clinicians and non-clinicians or between medical specialties. Clinicians who were no longer research active tended to emphasise the same negative factors as others, albeit more strongly.

## Discussion

The funding of clinical trials has become more transparent in recent years, due largely to ethics committee requirements and clinical governance. Payments for clinical involvement in both publicly and privately funded studies must flow to NHS employing organisations rather than individual clinicians. The requirement that full details of trial funding be made available to ethics committees not only increased transparency but made departures from recommended practice more difficult.

The findings on the factors motivating clinicians to participate in clinical trials provide UK evidence consistent with the few studies in other countries [9]. Interest in the research question, intellectual curiosity and potential benefits to patients were the most important factors. Payment for involvement was seen as less important and likely to have negative effects.

The boundary between paying and not paying for participation in clinical research is blurred by the acceptance by interviewees of the principle (and increasingly the practice) that the costs of research should be reimbursed. Expenses however are difficult to define unambiguously as they can be limited to expenses incurred or extended to include payment for the opportunity cost of time, such as time taken 'off-work' (either within or without employed hours) or leisure. While economists would argue that the right price would be that which led to target recruitment within time, several interviewees suggested a test: "would they be comfortable making public how much they received per patient recruited?" If the answer was no, then the payment was too high.

In the future, differences in funding and costs may become clearer when the NHS hosts both commercial and non-commercial trials within a single structure. All future trials in the NHS will have to be run through clinical research networks, which already exist for key diseases (cancer, mental health, diabetes, stroke, elderly, children, neurodegenerative diseases) with a comprehensive network to include all other diseases. "A key characteristic of the NHS networks will be to support and conduct randomised controlled trials and well designed studies for commercial and non-commercial sponsors. This will include pivotal licensing studies undertaken for industry on a full cost recovery basis." [24].

While we believe the findings of this study are valid, limitations are inevitable. One concerns restricting the interview sample to researchers in receipt of NHS HTA programme funding. Against this, most of these had received funding from other bodies, particularly the Medical Research Council and some had been involved in trials for the pharmaceutical industry. More important perhaps, all agreed to be interviewed free of charge, which may have biased the sample towards those who do research for reasons other than money. It also proved difficult to achieve the sample of researchers who were no longer active.

## Conclusion

Payment of clinicians beyond expenses is perceived to be a less important motivating factor than researching important, salient questions, and facilitating research by reducing bureaucracy and delay.

The exploratory research reported here indicated topics which might usefully be further researched including collecting improved data on the use of different motivating factors (including payments) for both clinician and patient involvement in clinical trials, monitoring the effects of the EU directive on Good Clinical Practice on the progress of clinical trials, researching the role of collaborators in clinical trials, and finally experimenting with the most promising motivating factors by randomising them within the context of multicentre clinical trials.

## Abbreviations

HTA: Health Technology Assessment; NCCHTA: National Coordinating Centre for HTA

## Competing interests

The authors declare that they have no competing interests.

## Authors' contributions

JP designed the project, obtained funding and ethics committee approval. When he moved to another university in 2005 JR took charge and supervised the qualitative interviews. The interviews were carried out by CK and analysed by CK, SH and JR. JR drafted the reports of the project with comments from all other authors.
